# Up-regulation of chemokine receptor CCR4 is associated with Human Hepatocellular Carcinoma malignant behavior

**DOI:** 10.1038/s41598-017-10267-4

**Published:** 2017-09-28

**Authors:** Xi Cheng, Huo Wu, Zhi-Jian Jin, Ding Ma, Stanley Yuen, Xiao-Qian Jing, Min-Min Shi, Bai-Yong Shen, Cheng-Hong Peng, Ren Zhao, Wei-Hua Qiu

**Affiliations:** 1 Department of General Surgery, Ruijin Hospital, Shanghai Jiao Tong University School of Medicine, Shanghai, 200025 China; 2 Shanghai Institute of Digestive Surgery, Ruijin Hospital, Shanghai Jiao Tong University School of Medicine, Shanghai, 200025 China; 3 Department of General Surgery, Ruijin North Hospital Shanghai Jiaotong University School of Medicine, Shanghai, 201800 China; 4 Department of General Surgery, First Affiliated Hospital of Anhui Medical University, Hefei, 230022 China; 5 Biology chemistry major, University At Albany, New York, United States

## Abstract

Studies indicate that the chemokine receptor is responsible for poor prognosis of hepatocellular carcinoma (HCC) patients. In this study, we initially demonstrated that CCR4 is overexpressed in HCC specimens, and its elevation in HCC tissues positively correlates with tumor capsule breakthrough and vascular invasion. Although overexpression of CCR4 failed to influent proliferation of HCC cells *in vitro* apparently, the prominent acceleration on HCC tumor growth *in vivo* was remarkable. The underlying mechanism may be involved in neovascularization. Interestingly, different from effect on proliferation, CCR4 overexpression could trigger HCC metastasis both *in vitro* and *in vivo* also induced HCC cell epithelial-mesenchymal transition (EMT) as well. Then we identified matrix metalloproteinase 2 (MMP2) as a direct target of CCR4 which plays an important role in CCR4-mediated HCC cell invasion, which was up-regulated by ERK/AKT signaling. Positive correlation between CCR4 and MMP2 expression was also observed in HCC tissues. In conclusion, our study suggested that chemokine receptor CCR4 promotes HCC malignancy and facilitated HCC cell metastases *via* ERK/AKT/MMP2 pathway. These findings suggest that CCR4 may be a potential new diagnostic and prognostic marker in HCC, and targeting CCR4 may be a potential therapeutic option for blocking HCC metastasis.

## Introduction

HCC is one of the most common cancers in the world, accounting for nearly half a million deaths worldwide^1–3^. Despite recent progress in diagnostic and therapeutic management, hepatocellular carcinoma (HCC) patient prognosis remains poor^4^. Radical surgical resection of the tumor is considered the only effective treatment for liver cancer, and provides a 5-year survival rate of 31%^5^. Frequent intrahepatic or pulmonary metastasis makes predominant contributions to high recurrence and mortality rate of HCC^6^. However, molecular mechanisms underlying HCC metastasis still remains largely unknown. Epithelial-mesenchymal transition (EMT) and matrix metalloproteinases (MMPs) appear to be a key factor that is often activated^7^.

Chemokines belong to a superfamily of small molecules (8–14 kDa), which could be classified into the four subfamilies: CXC, CC, C, and CX3C based on the sequence of conserved N-terminal cysteine residues^8^. Many studies have proven that chemokines and chemokine receptors are frequently associated with tumor metastases, such as CXCR4, CCR7 and CCR10 in breast and gastric cancer^9–11^, CXCR1 and CXCR5 in pancreatic cancer^12^. Several studies demonstrate that CXCR4 appears to be a major metastasis-regulating receptor while utilizing the lymph node trafficking network of hematopoietic progenitors and endothelial cells^13^. Although CCR4 expression in HCC has not been reported so far, the presence of CCL22, a CCR4 ligand, in tumor tissues and blood of patients with HCC has previously been reported in some studies^14^.

In this study, we initially demonstrated that CCR4 is overexpressed in HCC specimens, and its elevation in HCC tissues positively correlates with tumor capsule breakthrough and vascular invasion. It could also predict a poorer prognosis. Although overexpression of CCR4 failed to influent proliferation of HCC cells *in vitro* apparently, the prominent acceleration on HCC tumor growth *in vivo* was remarkable. The underlying mechanism may be involved in neovascularization. Interestingly, different from the effect on proliferation, we also noticed that CCR4 overexpression could trigger HCC metastasis both *in vitro* and *in vivo*. This might result from ERK/AKT/MMP2 pathway and EMT induction. These results may partially reveal possible molecular mechanisms of CCR4 promoting proliferation, migration, angiogenesis and invasion in HCC cells.

## Results

### CCR4 expression is unregulated in HCC tissues and predicts poor prognosis of HCC patients

**Figure 1:**
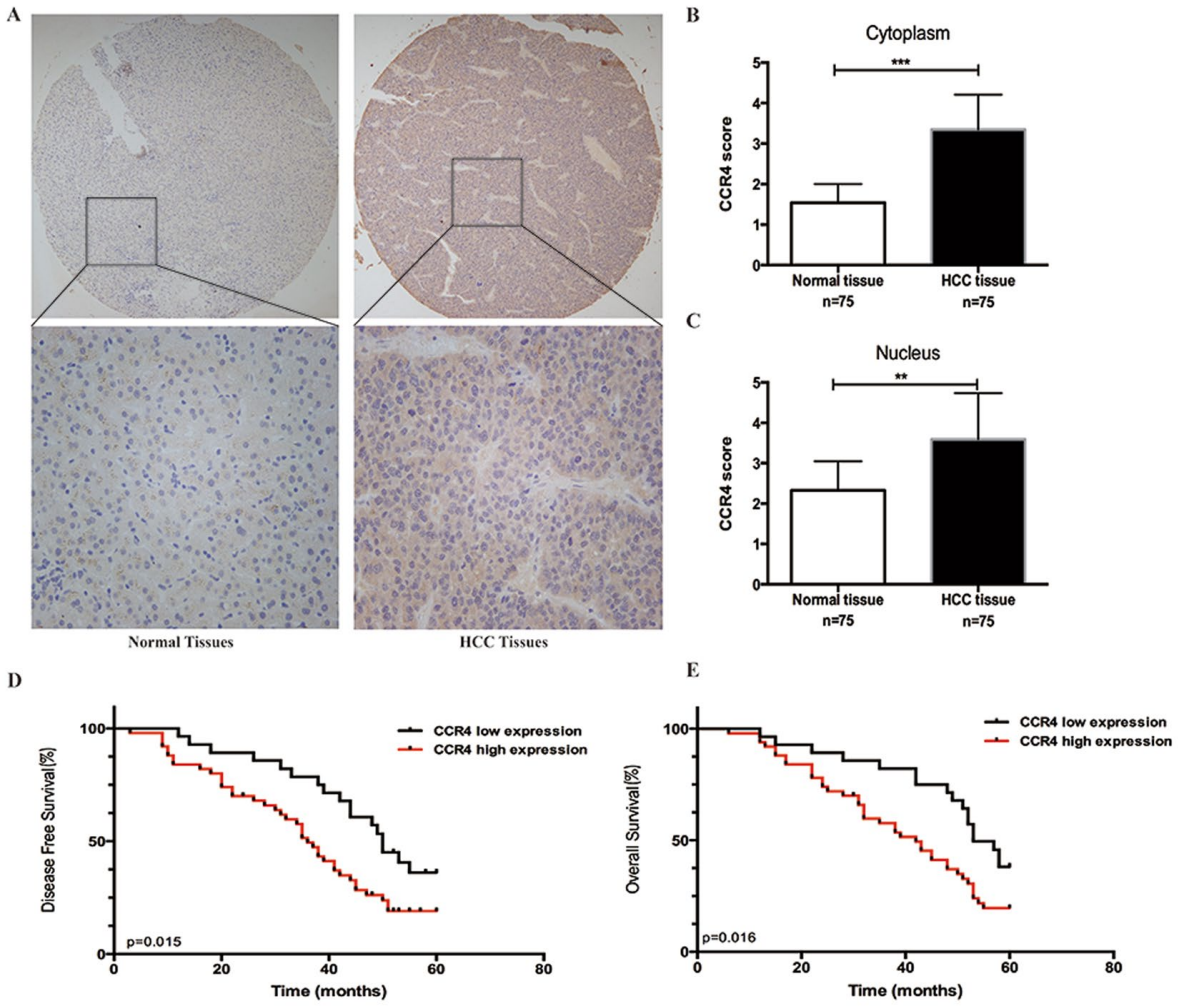
Immunohistochemistry to determine expression of CCR4 and its clinical significance in HCC patients. (**A**) CCR4 expression level in HCC tumor tissues and the paired normal tissues evaluated by immunohistochemical staining with tissue microarray. (Upper) 20×; (lower) 200×. (**B** and **C**) CCR4 scores based on the cytoplasmic or nuclear levels of expression in 75 HCC patients, compared with matched normal tissues. (**D** and **E**) HCC patients with high expression of CCR4 presented with worse overall survival, and disease free survival compared with that of low expression of CCR4. *p < 0.05, **p < 0.03, ***p < 0.01. Data represent the mean ± SD and are representive of three independent experiments.

We initially examined the expression of CCR4 in 75-paired cases of HCC samples. Table 1 summarized the patients’ demographics, pathological factors, and CCR4 expression. The ages of the patients ranged from 27 to 83 years with a median age of 57.26 years. Ninety-one percent were men (68 of 75) and 9% were women (7 of 75). The percentage of HBsAg positive status was 84% (63 of 75). IHC staining of 75-paired tissues indicated that CCR4 expression was significantly higher in HCC tissues than in the non-tumor tissues (Fig. 1A,B,C). The analysis showed that CCR4 expression could be detected in all 75 cases of HCC samples. In these cases, CCR4 positive expression was detected in 47 (62.7%) of the tumor tissues (*P* = 0.041, Table 1). Furthermore, we used HCC tissue microarray section to examine the correlation between CCR4 expression and clinicopathologic features, and found there was high expression of CCR4 which is associated with more vascular invasion (P = 0.001), and TNM stage (P = 0.013), but not with other clinicopathological factors including sex, age, cirrhosis and tumor diameter etc. (Table 1). In addition, we examined the correlation between CCR4 expression and clinic pathological features, and results were summarized in Table 2. A significant correlation was observed between cancer differentiation (P < 0.01) and nuclear grade (P < 0.01).

**Table 1:** Clinicopathological data.

Parameters	Case nubmber	CCR4 expression		P
		Low (28)	High (47)	
Gender				
Male	68	25	43	0.667
Female	7	2	5	
Age				
≤55	47	19	28	0.473
>55	28	9	19	
HBsAg				
Positive	63	24	39	0.755
Negative	12	4	8	
Family history				
Yes	21	8	13	0.932
No	54	20	34	
Cirrhosis				
Yes	73	27	46	0.707
No	2	1	1	
Tumor diameter (cm)				
≤5.0	31	11	20	0.781
>5.0	44	17	27	
Child-pugh class				
A	57	22	35	0.805
B	17	6	11	
C	0	0	0	
TNM stage				
I + II	60	26	34	0.032
III + IV	15	2	13	
Vascular invasion				
Yes	41	8	33	0.001
No	34	20	14	
Pathological stage				
I + II	58	26	32	0.13
III + IV	17	2	15

**Table 2:** Relationship between CCR4 expression, HCC Differention, and Nuclear Grade.

	CCR4 expression				
	0	+	++	+++	
Differentiation					
Well	6	3	2	2	P < 0.01
Moderate	2	6	6	6	
Poor	1	9	18	14	
Nuclear grade					
1	1	1	0	1	P < 0.01
2	6	20	4	1	
3	2	11	16	7	
4	0	0	1	4	

To further explore the clinical prognostic significance of CCR4 expression, Kaplan-Meier survival curve was used to evaluate the relation of CCR4 expression in HCC tissues with the survival time of HCC patients. The survival curve analyses showed that patients with high CCR4 expression (47 of 75, IHC staining shows uniformly positive) had shorter 5-year overall survival (OS) and disease free survival (DFS) than patients with low expression (28 of 75, IHC staining shows uniformly negative and heterogeneous intensity) (P < 0.05; Fig. 1D,E). The data of multivariate analysis by Cox proportional hazards models suggested that, however, CCR4 expression was not a significant independent prognostic risk factor (p > 0.05). These findings suggest that the up-regulation of CCR4 plays a critical role in HCC development.

### CCR4 expression does not affect the proliferation of HCC cells *in vitro*

**Figure 2:**
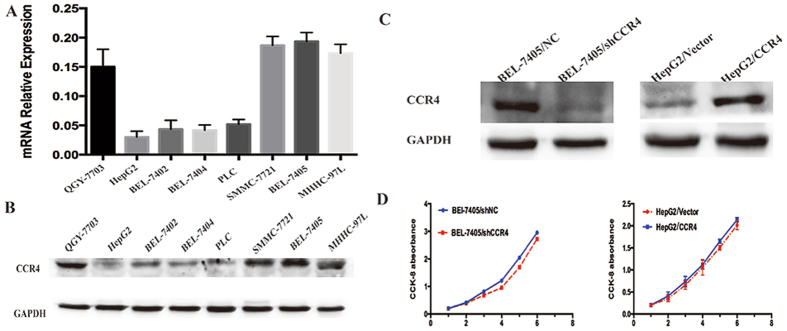
CCR4 expression does not affect the proliferation of HCC cells *in vitro*. (**A** and **B**) CCR4 expression level in eight HCC cell lines detected by qRT-PCR or western blot. (**C**) *Left panel*: effect of shCCR4 on CCR4 expression in BEL-7405 cells compared with shNC detected by western blot; *Right panel*: ectopic expression of CCR4 in HepG2 transfected with lentivirus-CCR4 and lentivirus-Vector detected by western blot. (**D**) Proliferation curve doesn’t show significantly effect on HCC cell growth for CCR4 down-regulate group or CCR4 up-regulate group. Data represent the mean ± SD and are representive of three independent experiments.

In order to explore particular role of CCR4 in HCC cells, we analyzed the expression of CCR4 in eight HCC cell lines by western blot and qRT-PCR. The results showed that CCR4 was highly expressed in BEL-7405 and QGY-7703, and lowly expressed in HepG2 and BEL-7405 (Fig. 2A,B). Therefore, we generated HepG2 cell line ectopically overexpressing CCR4 and employed lentivirus-mediated shRNA to down-regulate the expression of CCR4 in BEL-7405, respectively. The establishment of RNA interference and up-regulation stable clones was confirmed by Western blot and qRT-PCR (Fig. 2C).

Then, CCK-8 proliferation assay was performed to verify the influence of CCR4 on HCC cell lines proliferation *in vitro*. HCC cells expressing high level of CCR4 (HepG2/CCR4) failed to show significant higher proliferation potential when compared with wild type (HepG2/CCR4 vs HepG2/Vector, p > 0.05). On the contrary, interference of CCR4 expression (BEL-7405/shCCR4) also did not influent HCC cells proliferation apparently (BEL-7405/shCCR4, vs BEL-7405/shNC, p > 0.05). (Fig. 2D). Furthermore, similar results were observed in plant clone formation and Soft agar colony formation (Supplementary Figure 1).

### Enforcing CCR4 on HCC cells promotes tumor growth and micro-vessel density *in vivo*

**Figure 3:**
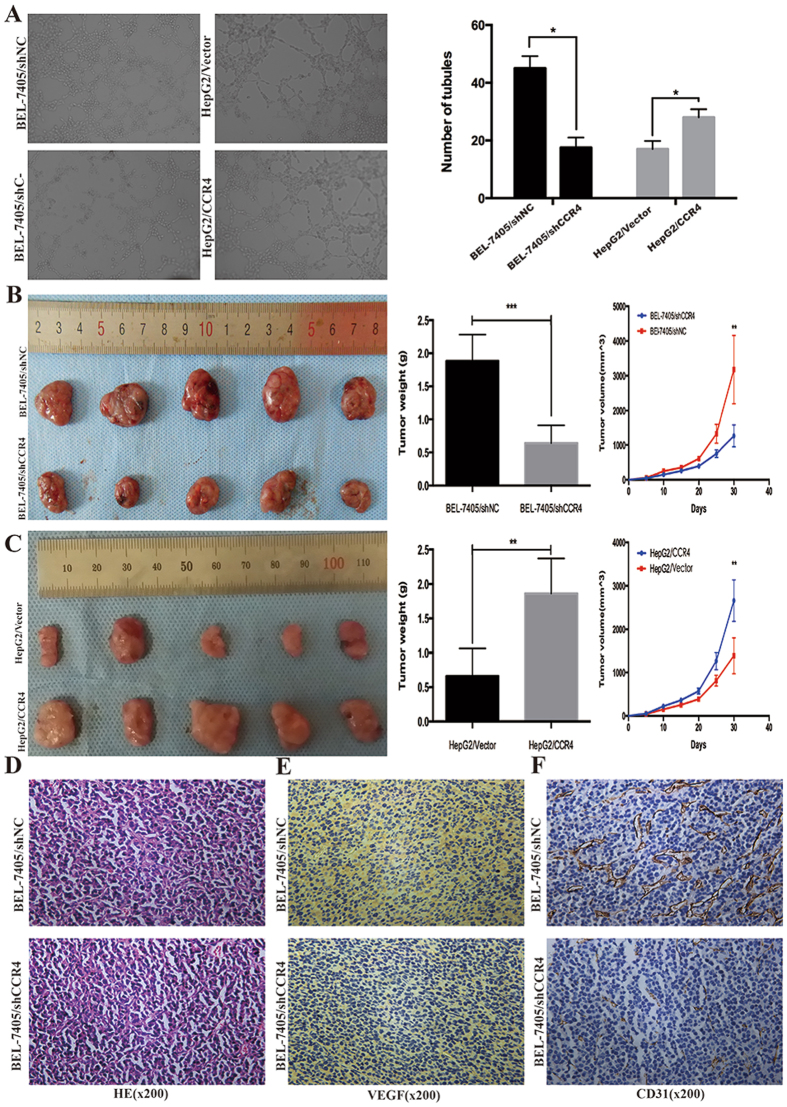
CCR4 facilitates HCC cells angiogenesis *in vitro* and *in vivo*. (**A**) Silencing CCR4 in BEL-7405 cells could significantly decrease the tubular formation ability of HUVEC while CCR4 overexpression could increase tubular formation ability significantly. (**B**) Volumes of CCR4 knockdown nude mice tumors were significantly smaller than those in control nude mice tumors. (**C**) Volumes of CCR4 overexpress nude mice tumors were significantly larger than those in control nude mice tumors. (**D**,**E** and **F**) Expression of tumor vasculogenic mimicry marker VEGF and CD31 were significantly decreased in CCR4 knockdown nude mice tumors. *p < 0.05, **p < 0.03, ***p < 0.01. Data represent the mean ± SD and are representive of three independent experiments.

To elucidate the role of CCR4 in tumor development *in vivo*, we established orthotropic xenograft tumor models. The stable clone expressing ectopic CCR4 over-express or CCR4 down regulate was subcutaneously injected into the flank of athymic nude mouse, and an equal volume of cells transfected with empty vector was injected into the opposite flank of the same mouse as the negative control. As shown in Fig. 3B, BEL-7405 cells stably silencing CCR4 formed smaller tumors than negative control group after 3 weeks’ observation (BEL-7405/shNC: 323.5 ± 112.5 mm^3^ *vs* BEL-7405/shCCR4: 125.6 ± 74.6 mm^3^ *p* < 0.05). As showing in Fig. 3C opposite results were showed in the CCR4 up-regulated group (HepG2/CCR4: 262.5 ± 87.4 mm^3^ *vs* HepG2/Vector: 147.3 ± 55.8 mm^3^ *p* < 0.05). The entire primary tumors were evaluated by pathologic examination. The pathologic results showed that microvessel density marker VEGF and CD31 tested by immunohistochemistry stained were significantly decreased in BEL-7405/shCCR4 group (Fig. 3D,E,F). These data might suggest that CCR4 could enhance HCC tumor growth *in vivo* by promoting blood vessel formation.

### CCR4 promotes HCC cells angiogenesis, migration and invasion *in vitro*

Tumor angiogenesis is a crucial aspect in the scenario of tumor growth and metastasis and, based on the data above, CCR4 could promote HCC tumor growth by facilitating blood vessel formation *in vivo*. To further elucidate the detailed mechanisms of CCR4 in tumor angiogenesis *in vitro*, human umbilical vein endothelial cell (HUVECs) was further included in tubular formation assay. Results showed that HepG2/CCR4 cells strong promoted tubular formation of HUVECs compared with control cells HepG2/Vector (Number of tubules: 31.4 ± 3.2 *vs* 18.7 ± 1.7, *P* < 0.05) (Fig. 3A). And we obtained opposite results after CCR4 was silenced (Number of tubules: 43.4 ± 5.7 *vs* 19.2 ± 1.4, *P* < 0.05) (Fig. 3A). Taken together, these results indicated that CCR4 was capable of manipulating the tumor angiogenesis ability of HCC cells *in vitro* and *in vivo*.

**Figure 4:**
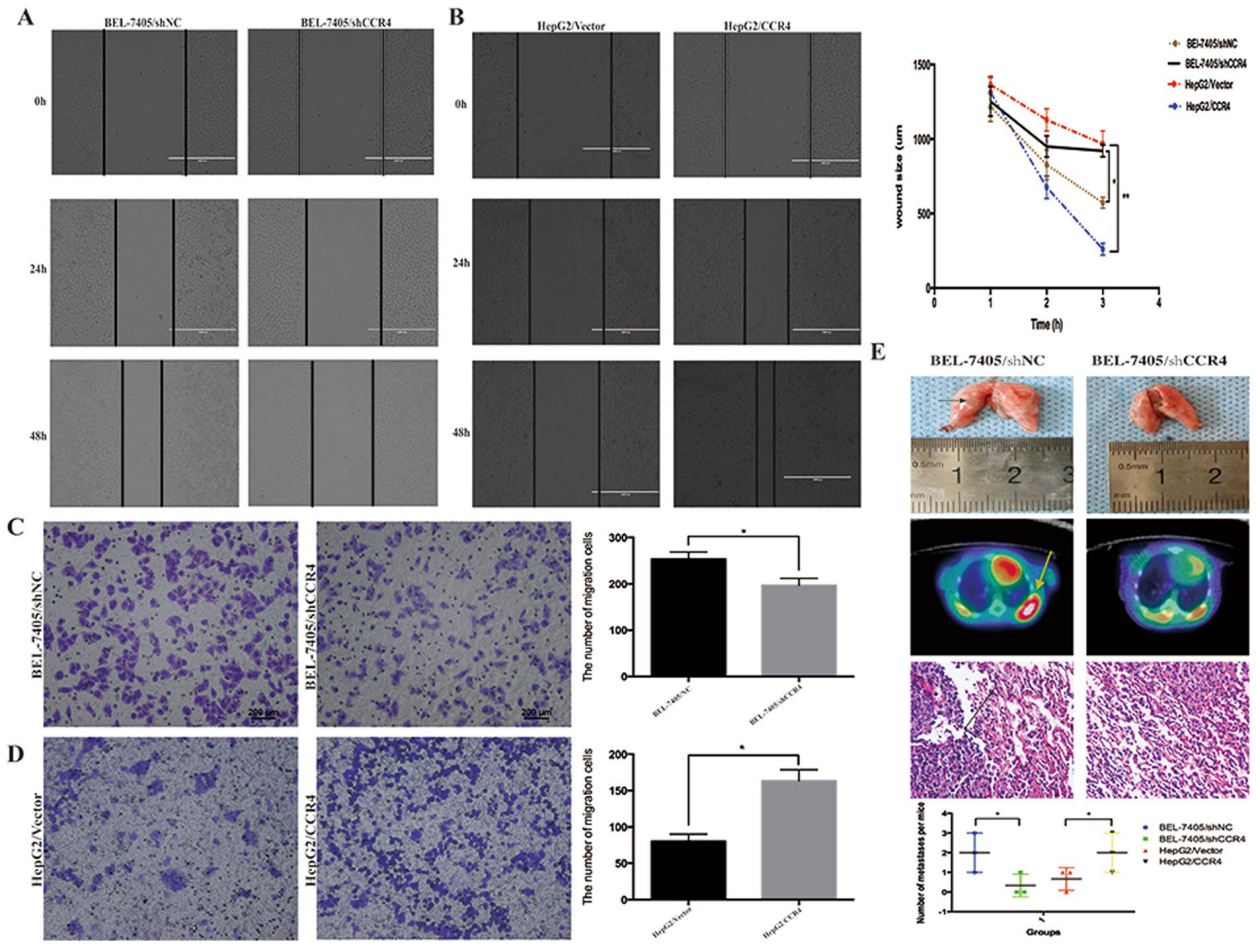
CCR4 promotes HCC cells metastasis *in vitro* and *in vivo*. (**A** and **B**) Wound-healing assay shows a significant decrease or increase in healing rate of the scramble wound in BEL-7405/shCCR4 and HepG2/CCR4 respectively. (**C**) Silencing CCR4 in BEL-7405 cells could reduce the migrated cells through transwell assay. (**D**) Overexpress CCR4 in HepG2 cells could significantly increase the migrated cells through transwell assay. (**E**) Typical image of the effect on lung metastases of HCC cells via tail vein injection. The arrows in the *upper panel* indicate lung metastasis tumors. Representative images of ^18^F-FLT micro-PET/CT images of mice are shown at the *middle panel*, arrow indicates ^18^F-FLT uptake positivity in thoracic metastatic lesions. While the pathological image showed in the *lower panel*, arrow indicate metastatic tumors. *p < 0.05, **p < 0.03, ***p < 0.01. Data represent the mean ± SD and are representive of three independent experiments.

In view of significant correlation between expression level of CCR4 and clinical invasive characteristics in HCC patients, CCR4 might play a positive role in HCC tumor metastasis. Wound healing assay was employed to detect the effect of CCR4 expression on migration ability of HCC cells. The results in Fig. 4A,B showed that down-regulated CCR4 could significant expand the distance between wound edges in BEL-7405/shCCR4 cells compared to BEL-7405/shNC cells (*P* < 0.05). Consistently, shorter distance could be observed in wound healing after overexpressing CCR4 in HepG2 cells (*P* < 0.05) (Fig. 4A and B).

Transwell assay showed that increased level of CCR4 promoted invasive abilities of HepG2 cells (HepG2/Vector: 82.6 ± 7.3, HepG2/CCR4: 172.4 ± 14.5, P < 0.05) (Fig. 4C), Invasive potential was dramatically impaired in BEL-7405/shCCR4 cells compared to BEL-7405shNC cells (BEL-7405/shCCR4: 195.2 ± 13.6, BEL-7405shNC: 267.5 ± 23.4, *P* < 0.05) (Fig. 4D).

### CCR4 promotes HCC cells metastasis *in vivo*

Based on the results above, *in vivo* model was employed to further confirm the enhancing effect of CCR4 on tumor metastasis. BEL-7405/shNC and BEL-7405/shCCR4 cells (1 × 10^6^ cells) were injected *via* tail vein to observe long-distance tumor metastasis *in vivo*. After eight weeks of observation, lung metastasis was examined by Micro/PET-CT and pathological study. Intriguingly, 5/5 mice injected with BEL-7405/shNC developed metastatic lung tumors with larger and greater numbers of nodules, whereas 2/5 visible metastatic tumors were found in BEL-7405/shCCR4 mice (Fig. 4E, P < 0.05). Histological analyses also confirmed the presence of lung metastases in these mice (Fig. 4E). All these results supported that CCR4 positively regulates metastasis of HCC cells both *in vitro* and *in vivo*.

### CCR4 induces EMT (epithelial-mesenchymal transition) in HCC cells

**Figure 5:**
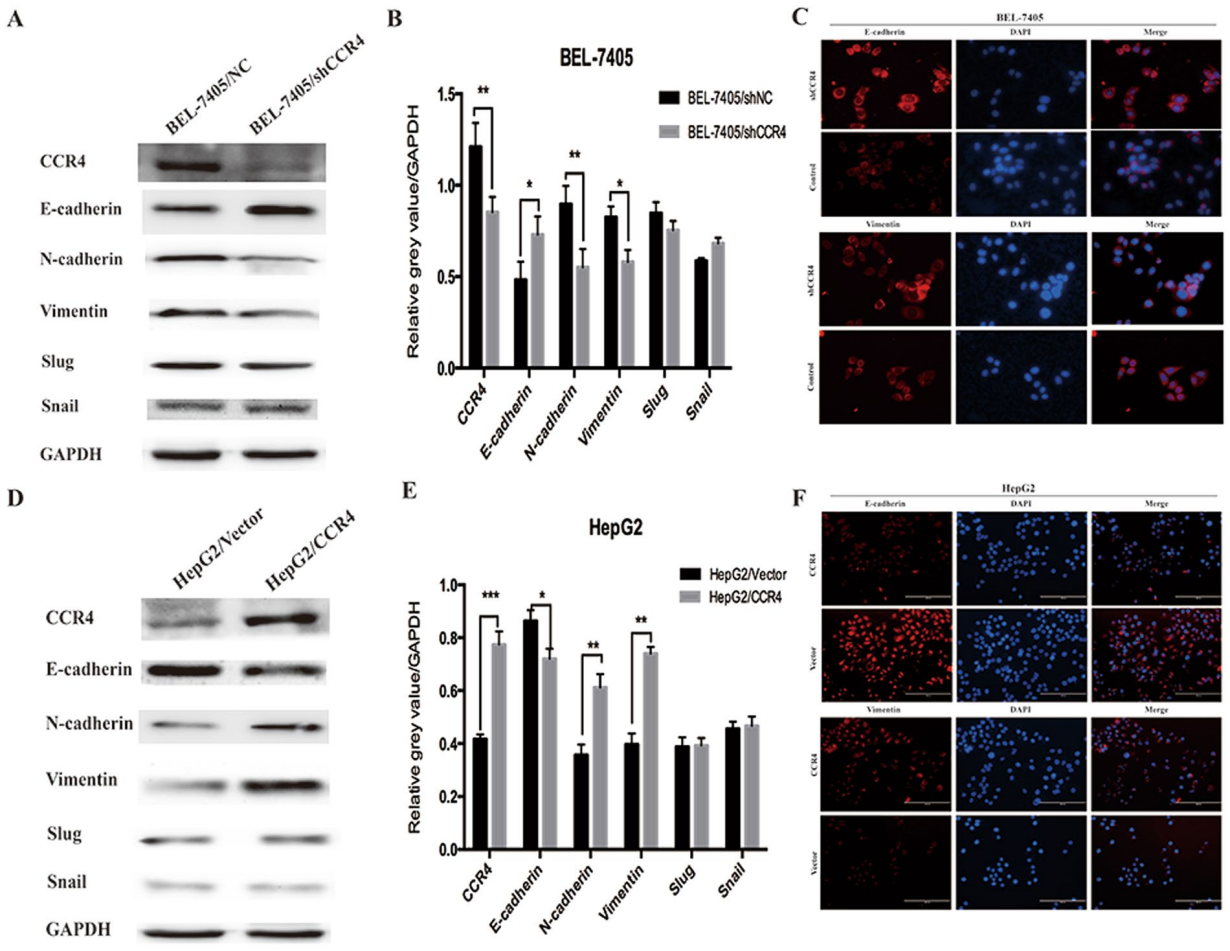
CCR4 induces Epithelial-mesenchymal transition (EMT) in HCC cells. (**A** and **B**) Western blot analyses showed that knockdown CCR4 in BEL-7405 cells could significantly decrease the expression of E-cadherin and increase the expression of N-cadherin and Vimentin. (**C**) Immunofluorescent staining showed that changes in EMT marker expression: E-cadherin expression is increased while Vimentin expression is decreased in BEL-7405/shCCR4 cells. (**D** and **E**) Western blot analyses showed that overexpress CCR4 in HepG2 cells could significantly decrease the expression of E-cadherin and increase the expression of N-cadherin and Vimentin. (**F**) Immunofluorescent staining showed that changes in EMT marker expression: Vimentin expression is increased while E-cadherin expression is decreased in HepG2/CCR4 cells. Data represent the mean ± SD and are representive of three independent experiments.

EMT-related proteins in HCC cells were evaluated by Western blot analysis. We observed that E-cadherin were increased in CCR4 down regulated cells, while N-cadherin, Vimentin protein level were decreased compared with their respective control cells (Fig. 5A,B). Converse results were obtained after CCR4 was over expressed in HepG2 cells (Fig. 5D,E). These results suggested a functional role for CCR4 in regulating EMT in HCC cells. Moreover, these observations in western bloting were similar with the findings in Immunofluorescence assay (Fig. 5C,F).

### MMP2 is responsible for CCR4-mediated HCC cells malignant biological behavior

**Figure 6:**
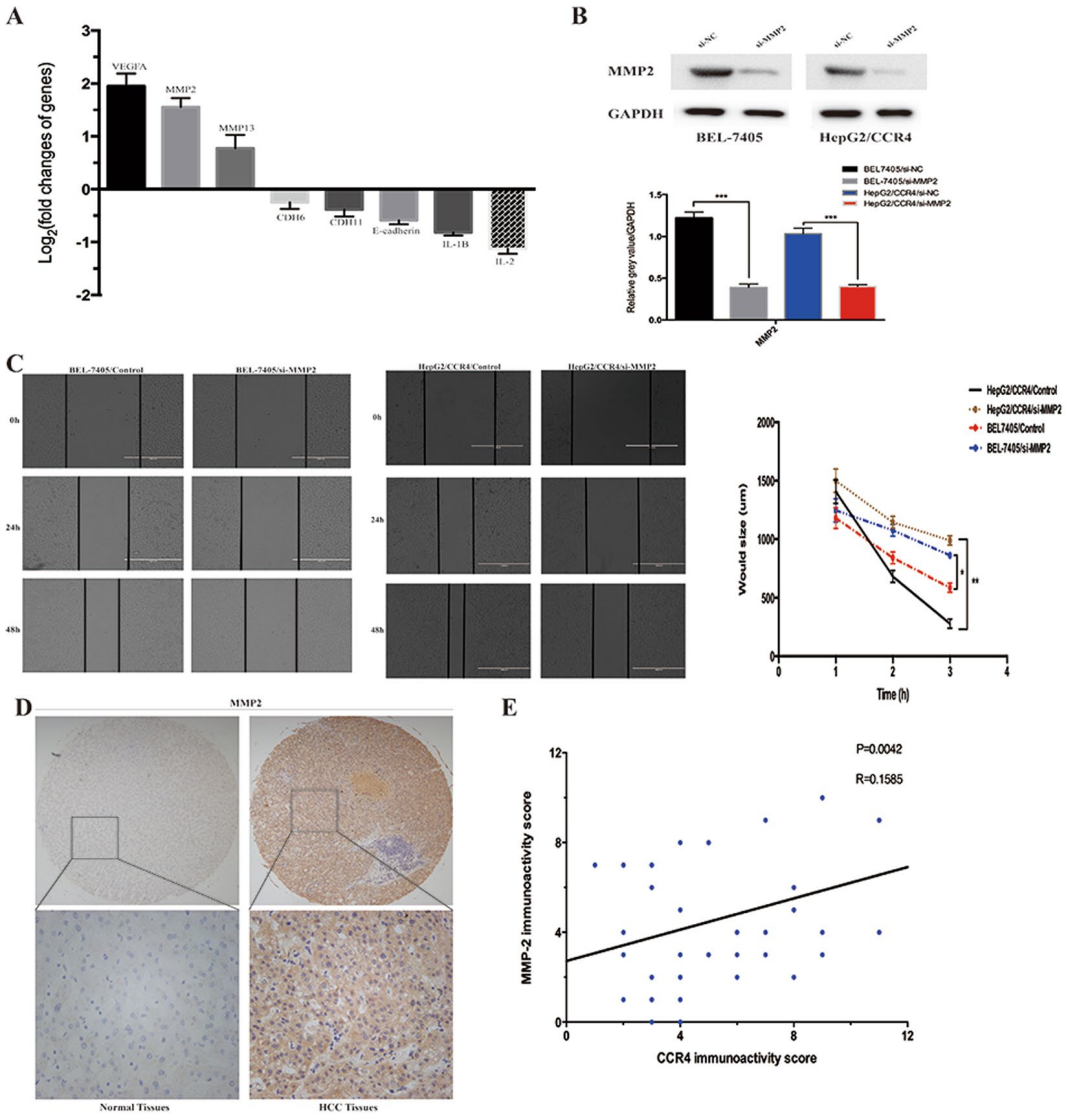
MMP2 plays a crucial role in HCC cells invasion mediated by CCR4. (**A**) Eight metastasis-related genes showed a more than 2-fold mRNA differential expression in Tumor Metastasis PCR array. (**B**) Effect of shMMP2 on MMP2 expression in BEL-7405 and HepG2/CCR4 cells detected by western blot. (**C**) Wound-healing assay shows a significant inhibitory role of siMMP2 in healing rate of the scramble wound in BEL-7405 and HepG2/CCR4 cells respectively. (**D**) MMP2 expression level in 75 cases of HCC tumor tissues and the paired normal tissues evaluated by IHC staining. (Upper) 20×; (lower) 200×. (**E**) Expression correlation of CCR4 and MMP2 was analyzed in 75HCC patients using IHC. *p < 0.05, **p < 0.03, ***p < 0.01. Data represent the mean ± SD and are representive of three independent experiments.

To further discover the possible mechanisms involved in the facilitation of HCC cells metastasis by CCR4, Tumor Metastasis PCR array was performed (Fig. 6A). And through the results of PCR array, we identified MMP2 as a prime candidate target of CCR4. It is well known that extracellular matrix (ECM) degradation by matrix metalloproteinases (MMPs) is critical for tumor invasion and metastasis. Previous studies have shown a significant expression of MMP2, 8,9, and 13 in HCC metastasis. Thus, to investigate the crucial role of MMP2 in CCR4-mediated cell invasion, RNA interference was used to silence MMP2 expression in HepG2/CCR4 and BEL-7405 cells. The efficiency of inhibition by MMP2 siRNA (si-MMP2) were confirmed by Western blot (Fig. 6B). After si-MMP2 treatment, wound-healing assay showed that the distance between the wound edges of down-regulated MMP2 groups were significantly longer than control groups (*P* < 0.05).

Then, we evaluated the expression of MMP2 in 75 HCC samples by IHC staining. (Fig. 6D). As expected, MMP2 was over-expressed in tumor samples compared to no-tumor samples (72.0% *vs* 22.6%, *P* < 0.001, Table 3). Furthermore, our results demonstrated a positive correlation between CCR4 expression and MMP2 (Pearson’s correlation, r = 0.1585, *P* < 0.005, Fig. 6E).

**Table 3:** The expression level of MMP2 in 75 HCC specimens.

Variable	Tissues (n = 75)		*P* value
	Carcinoma	Normal tissues	P < 0.01
MMP2 expression			
Positive	57	17	
Negative	18	58	

### CCR4 upregulates MMP2 via ERK/AKT pathway in HCC

**Figure 7:**
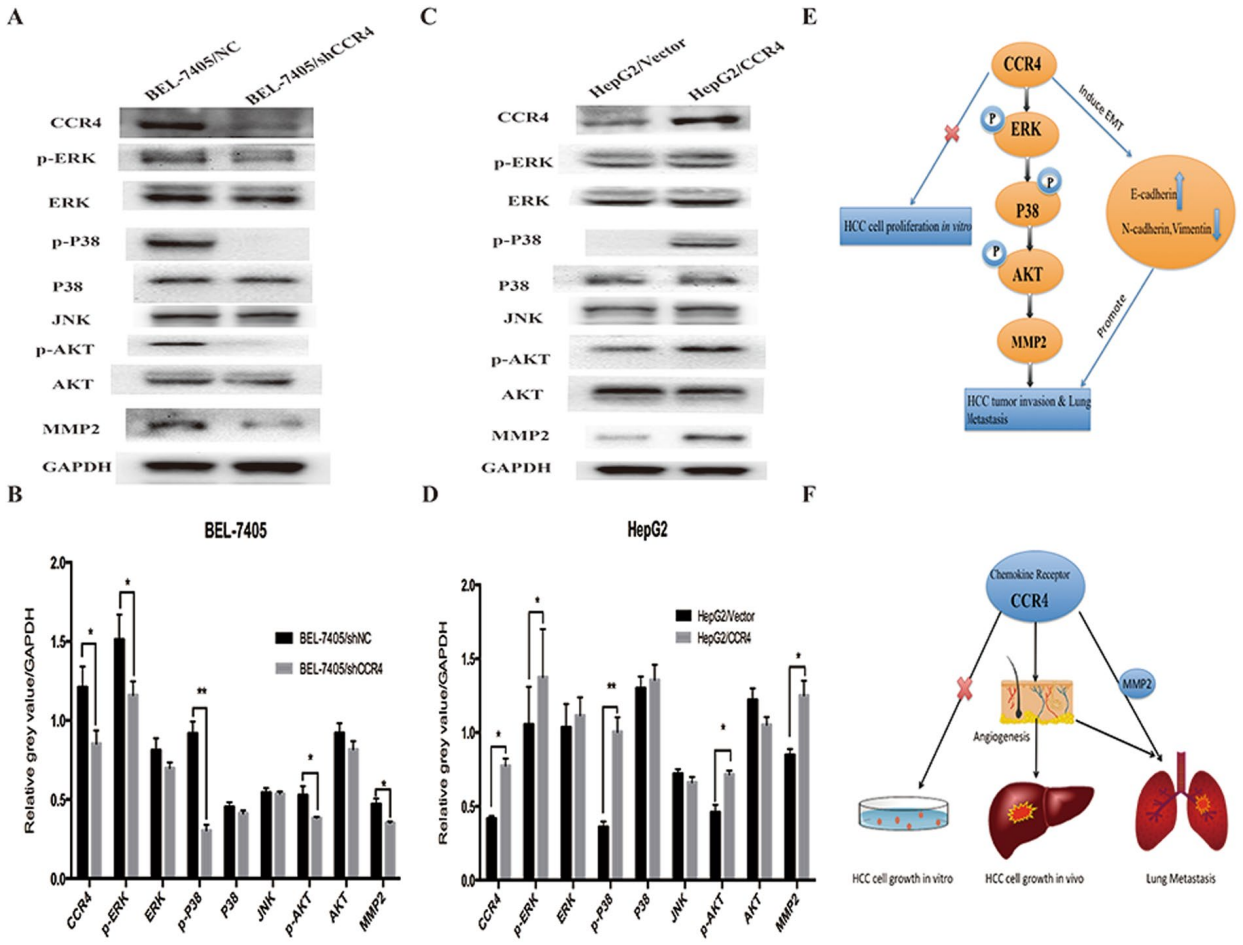
CCR4 up-regulates MMP2 expression through ERK/MAPK/AKT signaling pathway. (**A**) Effect of shCCR4 on p-ERK, p-P38, p-AKT, JNK and MMP2 expressions in BEL-7405 cells were detected by western blot. (**B**) Densitometry represents the expression of the proteins relative to GAPDH. (**C**) Effect of overexpress CCR4 on p-ERK, p-P38, p-AKT, JNK and MMP2 expressions in HepG2 cells were detected by western blot. (**D**) Densitometry represents the expression of the proteins relative to GAPDH. (**E** and **F**) Schematic representation of the effect of CCR4 facilitates HCC tumor biological behavior. Data represent the mean ± SD and are representive of three independent experiments.

Chemokines have been shown to associate with cancer metastasis as well as tumor angiogenesis, and work by activating some signaling pathways, including extracellular regulated protein kinases (ERK), phosphatidylinositol 3-kinase (PI3K)/protein kinase B (Akt), and phosphoinosmde-3-kinase (PI3K)/the mammalian target of rapamycin (mTOR). In this study we have already confirmed that CCR4 could promote HCC metastasis and angiogenesis *in vivo* and *in vitro*. Thus, we would like to elucidate the possible signal mechanisms involved in CCR4-mediated up-regulation of MMP2. We observed that overexpressed CCR4 in HepG2 increased the expression of phosphorylation of ERK, phosphorylation of AKT, phosphorylation of P38, compared with control groups (Fig. 7C,D). And opposing results could be observed in CCR4-silenced groups compared with control groups (Fig. 7A,D). Collectively, these findings suggested that CCR4 promote cancer metastasis and tumor angiogenesis through the up-regulation of MMP2 *via* ERK/AKT pathways in HCC (Fig. 7E,F).

## Discussion

Less than 20% of liver cancer patients are diagnosed when surgery is still an option. Of those who undergo surgery, the relapse rate remains as high as 50% in 5 years^15^. Total hepatectomy with liver transplantation may benefit a select group of patients not amenable to partial hepatic resection. However, the rate of recurrence may be as high as 50%^16^. Frequent pulmonary or intrahepatic metastasis makes predominant contributions to high recurrence and mortality rate of HCC. Therefore, it is worth exploring critical mechanisms, which may trigger HCC metastases. Many studies have already proven that chemokines and their receptors are closely associated with tumor growth and metastasis^17–19^. In this study, we demonstrated that the chemokine receptor CCR4 could not only promote HCC growth *in vivo* but also promote HCC metastasis *in vitro* and *in vivo*. Furthermore, we found that CCR4 was up regulated in HCC tissues and associated with poor prognosis of HCC patients. And we also found a remarkable correlation between the expression of CCR4 and tumor TNM stage, vascular invasion indicating that HCC cells expressed high level of CCR4 might have more invasive phenotype. These results showed that CCR4 might play a critical role in HCC.

Different from previous reports, in this study, we could not find any significant difference in proliferation ability between CCR4 knockdown cells BEL-7405/shCCR4 and control groups BEL-7405/shNC *in vitro* study. However, *in vivo* study, the significant inhibition of tumor could be observed by CCR4 knockdown in xenograft tumors model. Similarly, CCR4 overexpression in HepG2 cells (HepG2/CCR4) may lead to acceleration of tumor growth in xenograft model compared with HepG2/Vector cells. The significant differences in HCC cells proliferation by CCR4 between *in vitro* and *in vivo* studies in our research are consistent with Lee’s^20^ and Li’s^21^ findings in gastric cancer cells and breast cancer cells. Proliferation of tumors depends on multiple complex microenvironment factors, and some factors only play a role *in vivo* but not show their effect *in vitro*. The mechanism by which CCR4 promotes tumor growth *in vivo* still remains uncertain.

Tumor angiogenesis is a crucial aspect in tumor proliferation and metastasis particularly when tumors diameter approximates 2 mm^22^. It has been reported that chemokine receptors play an important role in tumor angiogenesis, such as CXCR1 and CXCR2^23,24^. CCR4 and its ligands contributed to vascular structure formation through the mobilization of smooth muscle precursor cells^25^. In this study, we demonstrated that micro-vessel density was decreased significantly in nude mice xenograft tumors implanted by CCR4–knockdown BEL-7405/shCCR4 cells, while we obtained opposite results in CCR4-overexpressing cells. Then, as a specific model for angiogenesis *in vitro*, HUVEC cells tubule formation also confirmed that CCR4 did promote angiogenesis. Thus, all these results suggested that chemokine receptor CCR4 played a key role in stimulating HCC growth through neovascularization *in vivo*, and in facilitating HCC angiogenesis both *in vitro* and *in vivo*.

It has been reported that the high expression of CCR4 level can promote tumor metastasis in lung cancer and breast cancer^21^. CCR4 could facilitate breast cancer metastasis by T cells down-regulation, and primary tumors can enhance the production of CCR4 ligand in the lungs of nude mice, which makes the CCR4 positive breast cancer cells migrate easily^26^. In our study, up-regulated CCR4 elevated the invasion of HCC cells significantly *in vitro* and faciliciated distant lung metastasis *in vivo*. Down-regulated CCR4 can consistently decrease the invasive capacity of HCC cells *in vitro* and *in vivo*. Our results herein suggested that CCR4 might function importantly in HCC metastasis.

Epithelial-to-mesenchymal (EMT) is a dynamic cellular process that is essential for the progression of cancer including tumor invasiveness, metastasis, senescence resistance, and apoptosis^27^. EMT always had a poor clinical prognosis in human cancers, and is characterized by the loss of epithelial features and the acquisition of mesenchymal phenotypes^28,29^. In our study, we found that silencing CCR4 could significantly decrease the expression of Vimentin and N-cadherin. Meanwhile the expression of E-cadherin was significantly increased, which might trigger the disruption of cell-to-cell adhesion. As expected, the opposing results could be observed in CCR4 up-regulated groups compared with control groups. All these results suggested that CCR4 could promote EMT in HCC cells in addition to metastasis.

Based on the results above, Tumor Metastasis PCR array was employed to further pinpoint the key molecular in CCR4 related metastasis. There were a total of 85 genes that met the analysis criteria for differential expression. Among them, VEGF and MMP2 represented the most overexpressed two moleculars, which could be the candidate targets of CCR4. The result of PCR array was confirmed by IHC in 75 HCC samples, and the expression of MMP2 in HCC tissue was significantly higher than normal tissues. Matrix metalloproteinases (MMPs) are a family of zinc endopetidases with proteolytic activity against the extracellular matrix components (ECM)^30^. MMP2, MMP9, and MMP13 are the main members of the MMPs family and had been reported to play a key role in tumor migration and remote metastasis in HCC and other cancers. In our current study, the expression of MMP2 could be closely related to down-regulateion of CCR4 in HCC cell lines. Vice versa, silencing MMP2 by siRNA could significantly suppresse CCR4-mediated invasion of HCC cells. All together, our results collectively indicated the remarkable positive correlation between MMP2 and CCR4.

Chemokines have been shown to work by activating several signaling pathways, including mitogen-activated protein kinase (MAPK), extracellular regulated protein kinases (ERK), phosphatidylinositol 3-kinase (PI3K)/protein kinase B (Akt), and the mammalian target of rapamycin (mTOR)^21^. CCR4 could facilitate metastasis via ERK/NF-кB/MMP13 pathway and acts as a direct target of TNF-α^19^. Our previous research also indicated that a Fibroblast growth factor receptor 3 novel aberrantly spliced transcript FGFR3_Δ7–9_ could facilitate HCC cell metastasis via ERK/MMP9 pathway^30,31^. Based on the positive interactive regulation between MMP2 and CCR4, we further confirmed the contribution of ERK and AKT pathways in HCC cell invasion. Thus, we concluded that CCR4 could advocate HCC cell invasion and induce EMT by activating ERK/AKT/MMP2 signaling pathway.

In conclusion, CCR4 could promote HCC malignancy, which can facilitate HCC tumor growth *in vivo* by neovascularization. It is noted that our data unravels a novel mechanism that CCR4 could accelerate metastasis and induce HCC cells EMT via ERK/AKT/MMP2 pathway. These findings suggest that CCR4 may be a potential new diagnostic and prognostic marker in HCC, and targeting CCR4 might be a promising strategy for HCC therapy.

## Materials and Methods

### Ethics statement

All procedures performed in studies involving animals were in accordance with the ethical standards and approved by the Animal Care and Use Committee of Ruijin hospital affiliated to Shanghai Jiaotong University School of Medicine (Permit number: CNIBR072809). All methods involving human participants were also performed in accordance with the relevant guideline and regulations and approved by Science and Technology Commission of Shanghai Municipality (Approval ID: RJXK 2012–0011).

### Patients and Tissue Specimens, and Cell Culture

From 2010 to 2011, 75 patients (68 males and 7 females), ranging from 31 to 81 years of age (mean age 56.6 years), were recruited in the current investigation with the informed consent and research consent approved by the Ethics Committee of Ruijin Hospital, Shanghai Jiao Tong University School of Medicine and all patients were fully informed of the experimental procedures. All 75 sets of HCC tissues and adjacent non-tumorous tissues (at least 5 cm away from the tumor margin) were collected from patients who underwent curative surgery at Ruijin Hospital. None of the patients had a history of radiotherapy or chemotherapy before surgery. Clinic pathological data were collected and pathological tumor staging was determined according to the UICC TNM classification. The histological types were assigned by at least three pathologists independently in a double-blinded fashion.

Human HCC cell lines HepG2 and PLC/PRF/5 were purchased from ATCC (Rockville, MD). Human HCC cell lines HCC-LM3, SMMC-7721, QGY7703, BEL-7404, and BEL-7405 were obtained from Institute of Biochemistry and Cell Biology, Shanghai Institutes for Biological Sciences, Chinese Academy of Sciences. Normal human fetal liver-derived cell lines HL-7702 were purchased from Institute of Biochemistry and Cell Biology as well. We cultured all cells in high glucose DMEM or PRMI 1640, 10% fetal bovine serum (FBS), and 1% P/S (100 IU/ml penicillin and 100 IU/ml streptomycin) at 37 °C and 5% CO2. Total RNA was extracted and isolated from tissue samples and cell lines using Trizol reagent (Invitrogen, Carlsbad, CA, USA). Reverse transcription of RNA was carried out using the reverse transcription kit (Promoga, USA).

### Real-time quantitative reverse transcription-PCR (qRT-PCR) and reverse transcription-PCR (RT-PCR)

Total RNA was isolated from cell lines and tissues using Trizol (Invitrogen) according to the manufacturers’ instructions. cDNA was synthesized by using reverse transcription kit (Invitrogen, CA). Quantitative polymerase chain reaction (PCR) was performed by using SYBR Green PCR Master Mix (Applied Biosystems, UK). The primers used were as follows: CCR4 sense primer 5′-GGG GTC ATC ACC AGT TTG-3′, CCR4 antisense primer 5′-TCT TCA CCG CCT TGT TCT-3′), GAPDH: 5′-GAA GGT GAA GGT CGG AGTC-3′, and 5′-GAA GAT GGT GAT GGG ATT TC-3′. Relative mRNA expression was calculated by comparative Ct method and GAPDH was used as the control. All experiments were done in triplicate. For RT-PCR, each cycle was carried out for 30 s at 95 °C, 30 s at 55 °C, and 60 s at 68 °C. Next, the products were separated electrophoretically in a 1.5% agarose and stained with DNA green. Images were acquired by Gel Doc EZ Imager (Bio-Rad, USA).

### Immunohistochemistry

Paraffin-embedded tissue sections from HCC specimens were given a heat pretreatment of 60 °C for one hour, then dewaxed in xylene, re-hydrated in an ethanol series (100–50%) and treated in 0.01 mol/L citrate buffer (pH 6.0) for antigen retrieval. After inhibition of endogenous peroxidase activity for 30 min with methanol containing 0.3% H_2_O_2_, the sections were stained with rabbit anti-CCR4 antibody (Abcam Biotechnology, dilution 1:300) at 4 °C overnight. The following experimental procedure was according to the manufacturer’s instructions of the LSAB + kit (Dako, USA). The results of immunostaining were determined by staining intensity and the number of positive cells (staining intensity: negative = 0, weak = 1, moderate = 2, strong = 3; and the percentage of cells stained: 0 = 0–1%, 1 = 1–5%, 2 = 6–29%, 3 = 30–59%, 4 = 60–100%). Three pathologists who were blinded from any patient data independently examined the cellular location of CCR4 and compared the staining between the tumor and normal tissues for each case.

### Scoring and Data Analyses

The diagnosis of HCC, tumor cell differentiation, the nuclear grade and growth pattern were assessed based on the examination of H&E-stained sections, according to the Edmondson’s grade and nuclear grade criteria. Briefly, well-differentiated HCC was composed of cells with minimal atypia and an increased nuclear/cytoplasmic ratio. In moderately differentiated HCC, tumor cells were arranged in trabeculae of three or more cells in thickness and had abundant eosphilic cytoplasm and round nuclei with distinct nucleoli. In poorly differentiated HCC, tumor cells have an increased nuclear/cytoplasmic ratio, frequent pleomorphism and bizarre giant cells. Nuclear grade was classified in a four-tiered system. Nuclear grade I HCC was comprised of adenoma-like cells that had abundant cytoplasm and showed little variation in size and shape and was closely similar to benign hepatocyte nuclei, with an even distribution of chromatin. Nuclear grade II HCC contained cells with large, prominent nucleoli, some degree of nuclear membrane irregularity, and chromatin clumping. Usually greater nuclear pleomorphism, angulated nuclei, and occasional multinucleated cells characterized grade III. Grade IV was noted for its distinct pleomorphism, hyperchromatism, and anaplastic giant cells.

### Immunoblot Analyses

As previously described^30^. 100 ug protein was separated by 10% SDS-PAGE gel and transferred to PVDF membranes. The membranes were blocked with 5% bovine serumalbumin (BSA) for 2 h and then were incubated at 4 °C overnight with primary antibodies. The primary antibodies for CCR4, MMP2 and GAPDH were purchased from Abcam, and antibodies against ERK, p-ERK, JNK, p-JNK, p38, p-p38, Akt, p-Akt, Vimentin, N-Cadherin and β-Catenin were purchased from Cell Signaling Technology, USA. Membranes were then incubated with secondary antibody for 2 h at room temperature and were visualized using an enhanced chemiluminescence detection system (Amersham Bioscience, Piscataway, NJ, USA) according to the manufacturer’s protocol. Three independent experiments were conducted at the same conditions.

### Immunofluorescence assay

For immunofluorescence assays, 30,000 cells were culturedin each cell of EZ slides (Millicell EZ SLIDE 8-well glass, Millipore). After being incubated for 24 h, each well was fixed in 4% paraformaldehyde for 15 min. Cells were permeabilized with 0.5% TritonX-100 for 20 min at room temperature, wash three times with phosphate-buffered saline (PBS), and then blocked with PBS containing 5% (w/v) bovine serumalbumin (BSA) for 1 h at room temperature. Cells were treated with anti-E-cadherin antibody (1:50, Cell Signaling Technology) and anti-Vimentin antibody (1:50, Abcam) and incubated overnight at 4 °C. A negative control (without primary antibody) was included on every slide. On the second day, each well was washed with PBS and incubated with iFluor™ 594 goat anti-mouse antibody (AAT Bioquest, USA) for 2 h at 37 °C. After being washed with PBS, diamidino-2-phenylindole (DAPI; Santa Cruz) was used to counterstain nucleus. Results were obtained from fluorescencemicroscopy (Olympus, Japan). All experiments were conducted in triplicates.

### Generation of gene overexpressing and knockdown stable cells

Green fluorescent protein (GFP)-labeled lentiviral vector and four short hairpin RNA (shRNA) targeting CCR4 were purchased from Genepharma Shanghai. We transfected Lentivirus particles were into the HCC cells according to the manufacturer’s instruction. 4 targeted CCR4 sequences were listed as follows: shRNA1: 5′-GGT TCT GGA CAC ACA CTT ACA-3′; shRNA2: 5′-GCA CCT TTG AAA ACT GAT TGG-3′; shRNA3: 5′-GGG AGA TTC GCA AAT AGT ACA-3′; shRNA4: 5′-GCA CAC CAT GGA GTC TGG ATC ATG A-3′. Lipofectamine 2000 (Invitrogen Corporation) was used to mediate shRNA and then transfected into cells. G418 were used to select the stable transfected cells. And plasmid shRNA2 proved to have strongest efficiency and we used this for further research.

### SiRNA transient transfection

We purchased MMP2 siRNA and control siRNA were from Genepharma, Shanghai. The cells were transfected with siRNA with Lipofectamine 3000 mediating according to the manufacturer’s instructions.

### Cell viability assay

Cell viability assay was evaluated by Cell Counting kit-8 (CCK-8; Dojindo Molecular Technologies Inc) assays. About 2000 cells were plated in each well of 96-well plates and cultured in 37 °C, 5%CO_2_ incubator. Four groups (BEL-7405/shNC, BEL-7405/shCCR4, HepG2/Vector and HepG2/CCR4) were designed and each group had six copies. The cell viability was detected at 0, 24, 48, 72, 96 and 120 h. To evaluate cell viability, 10-μL CCK-8 was added into each well and the plate was incubated at 37 °C for 2 h after then the absorbance at 450 nm was recorded by OD detection using spectrophotometer. Data came from three independent experiments.

### Colony Formation Assay

For colony formation assays, 1000 cells were plated onto 6-well plates and incubated at 37 °C for about 14 days to allow colonies to develop. After macroclones (large than 5 mm) were formed, the cells were washed twice with PBS and fixed in 4% paraformaldehyde for 30 min. Then, cells were stained with 1% crystal violet for 30 min, and the numbers of colonies per well were counted. Three independent experiments were conducted for the same conditions.

### Transwell migration and invasiNon assay

Cell migration ability of HCC cells was estimated by transwell assay using Falcon^TM^ Cell Culture Insert (BD353097) according to the manufacturer’s instructions. 1 × 10^5^ constructed HCC cell clones were suspended in serum-free RPMI-1640/DMEM and plated on transwell chambers. The medium containing 10% FBS was added to the lower chamber as chemoattractant. After 24 h, the chambers were stained with 1% crystal violet solution for 15 min and immersed in PBS for 10 min. Then, the cells in the lower chamber were observed and counted under an inverted microscope. The values are expressed as the mean cell numbers under five random fields of view (200×). Three independent experiments were conducted for the same conditions. Three independent experiments were conducted for the same conditions.

### Wound healing assay

Cells were seeded and cultured in six-well plates with a serum free medium for 24 h. Wounds were scratched on the monolayer of cells using 20 μL pipette tips. Fresh medium was then replaced. Plates were washed once with fresh medium to remove non-adherent cells after the cells had been cultured for 0, 12 or 24 h, and then photographed. Finally, the distances between wound edges were measured.

### Endothelial tube formation analysis

Endothelial tube formation analysis was employed for *in vitro* angiogenesis assay. HUVECs were cultured in tumor supernatant of each group in 96-well plate coatedwith 40 ul Matrigel (BD Bioscience) at a density of 3 × 10^4^ cells per well after Matrigel was polymerized at 37 °C for 1 h. After 6-h incubation at 37 °C with 5%CO_2_, tubules were photographed by microscopy andtubular numbers, length and intersections were countedaccording to published protocols by Image-Pro Plussoftware. Data came from three independent experiments.

### *In vivo* tumorigenesis and metastasis assays by Micro PET/CT

For tumorigenesis assays, HCC xenografts were established in nude mice. BEL-7405/shNC, BEL-7405/shCCR4, HepG2/Vector and HepG2/CCR4 (1 × 10^6^ cells) were subcutaneously injected into 4-week-old male BALB/c nude mice (Institute of Zoology, China Academy of Sciences). Tumor nodules were measured every 7 days, and were calculated using the formula: tumor volume = (Width^2^ × Length)/2. Mice were killed 6 weeks after injection. Tumors were weighed and fixed for immunohistochemistry staining. All animal research consent approved by the Ethics Committee of Ruijin Hospital, Shanghai Jiao Tong University School of Medicine

For metastasis assays, BEL-7405/shNC, BEL-7405/shCCR4, HepG2/Vector and HepG2/CCR4 (1 × 10^6^ cells) were injected into the tail vein of 4-week-old male BALB/c nude mice. After six weeks, the lung metastasis lesions were detected by micro-PET/CT. All of the suspicious lung metastasis sites were evaluated by histological examination after the mice were sacrificed. PET/CT imaging was performed on an Inveon MM Platform (Siemens Preclinical Solutions, Knoxville, Tennessee, USA) with a computer-controlled bed and 8.5 cm transaxial and 5.7 cm axial fields of view (FOV). The animals were anesthetized with 2% isoflurane in O_2_ gas for [18 F]-FDG injection (a single injection of 0.1 ml FDG with an activity of 10MBq intravenously in the tail vein), immediately awakened afterwards and placed back in the anesthesia cage. Two hours after administration of the tracer injection, animals were anesthetized with isoflurane, placed prone on the PET scanner bed near the central field of view and were maintained under continuous anesthesia during the study with 1.5% isoflurane in oxygen at 2 L/min. Inveon Acquisition Workplace (IAW) 1.5.0.28 was used for scanning process. Ten min CT X-ray for attenuation correction was scanned with a power of 80Kv and 500 μA and an exposure time of 1100 ms before PET scan. Ten-minute static PET scans were then acquired, and images were reconstructed by an OSEM3D (Three-Dimensional Ordered Subsets Expectation Maximum) algorithm followed by MAP (Maximization/Maximum a Posteriori) or FastMAP provided by IAW. The 3D regions of interest (ROIs) were drawn over the heart guided by CT images and tracer uptake was measured using the software of Inveon Research Workplace (IRW) 3.0. Individual quantification of the [18F]-FDG uptake in each of them was calculated. Mean standardized uptake values (SUV) were determined by dividing the relevant ROI concentration by the ratio of the injected activity to the body weight. Then, mice were killed and examined microscopically by H&E staining for the development of metastasis. All experiments were performed in accordance with the official recommendations of the Chinese animal community and animals received humane care according to the criteria outlined in the “Guide for the Care and Use of Laboratory Animals”.

### Statistical Analyses

An analysis of variance (ANOVA) and Student’s *t* test were used for comparison among groups. The Mann-Whitney U test was used for comparison of tumor volume. Categorical data was evaluated with *chi*-square test or Fisher exact test. A *p*-value less than 0.05 was considered to be significant.

## Electronic supplementary material

Supplementary Figure and Original data
