# Supplementary material for: Up-regulation of chemokine receptor CCR4 is associated with Human Hepatocellular Carcinoma malignant behavior
Source: Sci Rep. 2017 Sep 28;7:12362. doi: 10.1038/s41598-017-10267-4 (PMC5620046; doi:10.1038/s41598-017-10267-4)
Supplement: Supplementary file 1 — Supplementary Figure and Original data [file 41598_2017_10267_MOESM1_ESM.pdf]

Up-regulation of chemokine receptor CCR4 is associated with Human Hepatocellular Carcinoma malignant behavior

Xi Cheng[1,2]*, Huo Wu[1,3]*, Zhi-Jian Jin[1,2]*, Ding Ma[1,2], Bai-Yong Shen[1,2], Cheng-Hong Peng[1,2]**, Ren Zhao[1,2]**&Wei-Hua Qiu[1,2]**

Supplementary Figure1. CCR4 expression doesn't affect the proliferation of HCC cells in vitro.

A&B. Plant clone formation assay doesn't show significantly effect on HCC cell growth for CCR4 down-regulate group or CCR4 up-regulate group. **C&D**. Soft-agar clone formation doesn't show significantly effect on HCC cell growth for CCR4 down-regulate group or CCR4 up-regulate group

Supplementary Data of Figure2A; 5A&D; 6A&B; 7A&C are replicates rather than the original photography of the blots in the main figures, All these blots come from replicate experiments!

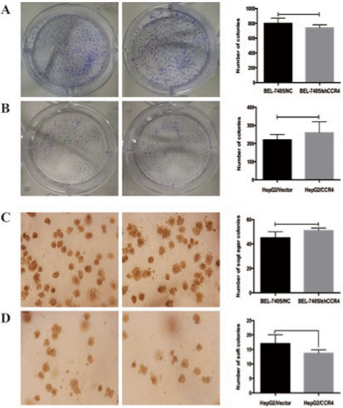

Supplementary Figure1. CCR4 expression does not affect the proliferation of HCC cells *in vitro*.
A&B. Plant clone formation assay doesn't show significantly effect on HCC cell growth for CCR4 down-regulate group or CCR4 up-regulate group.
C&D. Soft-agar clone formation doesn't show significantly effect on HCC cell growth for CCR4 down-regulate group or CCR4 up-regulate group.

Supplementary supporting data for Figure 2A. 5 A,D and 6 A,B

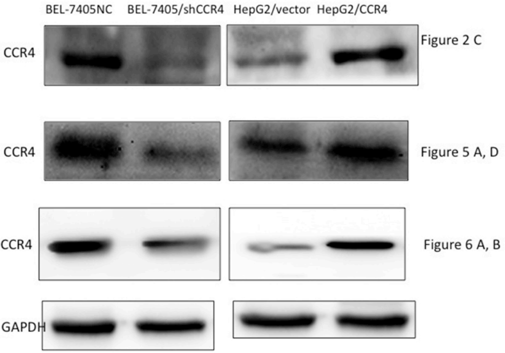

Supplementary supporting data for Figure 3A

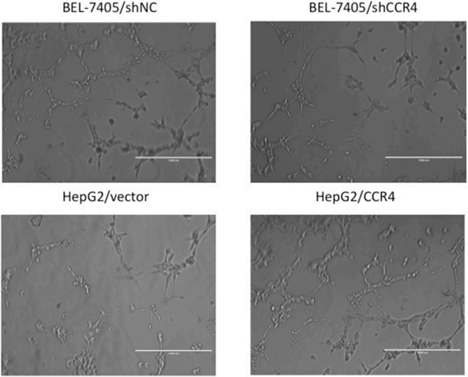

Supplementary supporting data for Figure 4A

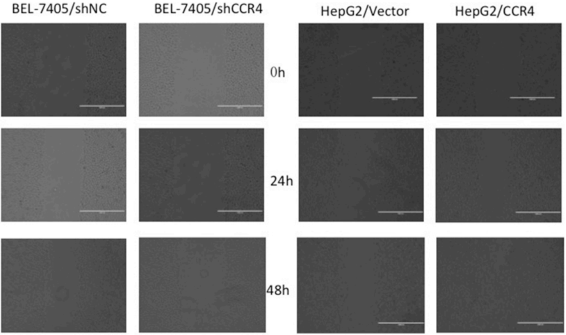

Supplementary supporting data for Figure 4B

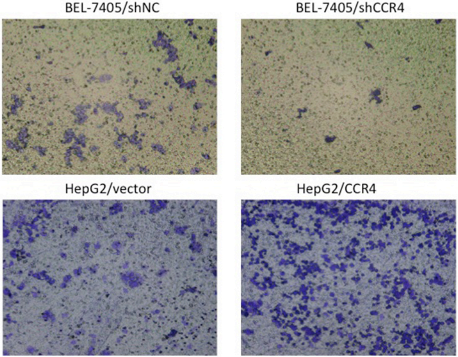

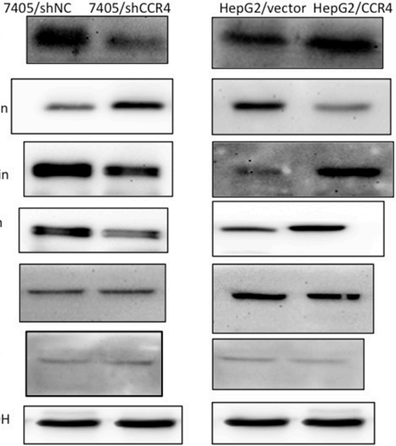

Supplementary supporting data for Figure 5A&D

Supplementary supporting data for Figure 6C

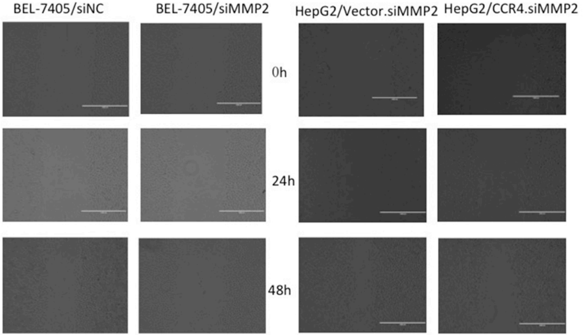

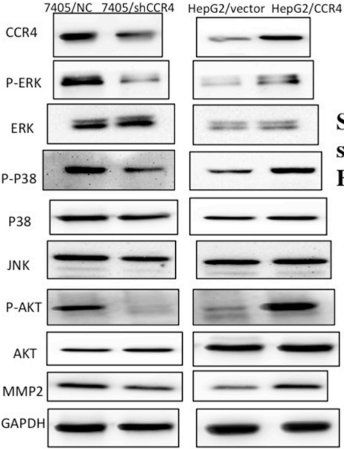

Supplementary supporting data for Figure 7.A&C
